# Global *Coffea arabica* variety trials reveal genotype-by-environment interactions in resistance to coffee leaf rust (*Hemileia vastatrix*)

**DOI:** 10.3389/fpls.2025.1583595

**Published:** 2025-06-13

**Authors:** Jorge C. Berny Mier y Teran, Solene Pruvot-Woehl, Catherine Maina, Santos Barrera, James Mwita Gimase, Brahim Banda, Albertino Meza, Nathan Aliel Kachiguma, Elijah K. Gichuru, Julio Alvarado, Suresh Kumar, Jonny Alonso Castillo, Beatriz Moreno Lopez, Ariana Karina Román Ruíz, Ari Wibowo, Never Mwatsiya, Jeena Devasia, Divya Kallingapuram Das, Edgardo Alpizar, Melanie Bordeaux, Miftahur Rizqi Akbar, Piet van Asten, Jean Baptiste Kayigamba, Paul Mulemangabo, Godfrey Sseremba, Benit Mate, Hans Alexander Méndez Mendoza, Simon Martin Mvuyekure, Jane Jerono Cheserek, Rosalío López Morgado, Chemutai Job Alunga, Nayani Suryprakash Rao, Samson Tarusenga, Tania Humphrey, Christophe Montagnon

**Affiliations:** ^1^ World Coffee Research, Portland, OR, United States; ^2^ TechnoServe, Arlington, VA, United States; ^3^ Kenya Agricultural & Livestock Research Organisation, Nairobi, Kenya; ^4^ OLAM, Kateshi site, Zambia; ^5^ Cooperativa CENFROCAFE, Jaen, Cajarmarca, Peru; ^6^ ECOM, Managua, Nicaragua; ^7^ Department of Agricultural Research Services, Mzuzu, Malawi; ^8^ Central Coffee Research Institute, Chikmagalur, Karnataka, India; ^9^ AGROLAV, Santa Ana, El Salvador; ^10^ Fundación NicaFrance, Finca La Cumplida, Matagalpa, Nicaragua; ^11^ Asociación Nacional del Café, Ciudad de Guatemala, Guatemala; ^12^ El Colegio de la Frontera Sur, Tapachula, Chiapas, Mexico; ^13^ Indonesian Coffee and Cocoa Research Institute, Jember, Indonesia; ^14^ Coffee Research Institute, Chipinge, Zimbabwe; ^15^ OLAM, Paksong site, Paksong, Lao People's Democratic Republic; ^16^ National Institute for Agronomic Study of the Belgian Congo, Mulungu, Democratic Republic of Congo; ^17^ National Coffee Resources Research Institute, Mpoma, Uganda; ^18^ Café Finca Mountain Villa Rica, Chanchamayo, Peru; ^19^ Rwanda Agriculture Board, Huye, Rwanda; ^20^ Instituto Nacional de Investigaciones Forestales, Agricolas y Pecuarias, Campo Experimental Cotaxtla, Veracruz, Mexico; ^21^ RD2 Vision, Valflaunes, France

**Keywords:** *Coffea arabica*, coffee leaf rust, resistance, multi-location trial, genotype by environment

## Abstract

**Introduction:**

Coffee leaf rust (CLR), caused by the obligate parasitic fungus *Hemileia vastatrix*, is the most significant constraint in Arabica coffee production worldwide. The disease is ubiquitous, and in severe infections, it can lead to defoliation of coffee plants, impacting yield and quality. The use of resistant varieties is the most cost-effective and sustainable strategy for managing coffee leaf rust. Identifying highly resistant varieties, as well as environments where these varieties perform similarly, is a crucial step in breeding programs.

**Methods:**

An international, multi-institutional effort involved the evaluation of 29 varieties, developed by different breeding programs in coffee-producing countries across the globe, for CLR severity under field conditions at 23 sites in Africa, Asia, and Latin America.

**Results:**

The results showed that both the genotype and genotype-by-site interaction were highly significant, indicating that resistance to coffee leaf rust depends not only on the genetic makeup but also varies between sites. In general, varieties with interspecific introgressions were more resistant than the nonintrogressed pure Arabicas. Although stability and overall resistance were correlated, some of the most resistant varieties were not the most stable. Four mega-environments were identified, and sites that were better at discriminating for resistance were found across the three continents.

**Discussion:**

Overall, this multi-institutional cooperation led to the identification of both locally and globally highly resistant coffee leaf rust varieties, as well as an understanding of their underlying genetics and the further causes of genotype-by-environment interactions concerning coffee leaf rust resistance.

## 1 Introduction

Coffee (*Coffea* spp.) is one of the most widely consumed beverages in the world, valued at USD17 billion at the farm level and a retail value of USD200 billion (ICO, 2020). As of 2022, coffee is grown in 82 countries on over 12.2 million Ha of land (FAOSTAT, 2024) and by over 12.5 million farm households worldwide (Montagnon et al., 2021). The coffee trade is the main source of income for more than 100 million people (Hoffmann, 2018; Talhinhas et al., 2017). Global coffee production is predominantly smallholder-driven, with smallholders cultivating less than 5 Ha supplying 60% of the global coffee market, and farms between 5 and 50 Ha producing another 19% of the world supply (Siles et al., 2022). The *Coffea* genus comprises 130 species (Davis and Rakotonasolo, 2021); however, only two species, *Coffea arabica* and *Coffea canephora*, are of global economic importance, contributing 67% and 33% of the total trade volumes, respectively (International Coffee Organization, 2025).

Coffee leaf rust (CLR) is one of the major limitations of global Arabica coffee production (Avelino et al., 2015; Zambolim, 2016), with estimated annual economic losses of about USD2 billion due to control costs (McCook, 2019; Van der Vossen et al., 2015; Zambolim, 2016). In 2016, the International Coffee Organization estimated economic losses of USD616 million in Central America as a result of coffee leaf rust, leading to a drastic decline in coffee prices (McCook and Vandermeer, 2015). Coffee leaf rust was first noticed in 1861 in Western Kenya, but its epidemic in commercial coffee was recorded in 1869 in Ceylon, now Sri Lanka (McCook, 2019). By the 1920s, it had managed to spread to most parts of Africa and Asia, and by 1985, the disease had spread to almost every coffee-growing region in the world (McCook and Vandermeer, 2015), except Hawaii, where it was eventually recorded in 2020 (Ramírez-Camejo et al., 2022).

Coffee leaf rust is caused by the fungal pathogen *Hemileia vastatrix* Berkeley and Broome (Talhinhas et al., 2017) and is an obligate coffee-specific pathogen characterized by a powdery coating of yellow urediniospores on the underside of the coffee leaves (Gichuru et al., 2021). The coffee leaf rust fungus, unlike the other plant pathogenic rust fungi that reproduce both sexually and asexually, has a complex life cycle that contains up to five different sporulating stages to complete the cycle (Talhinhas et al., 2017; Rhiney et al., 2021). It is dispersed by spores spread by wind, rain splash, insects, and animals, including humans (Kushalappa and Eskes, 1989; Avelino et al., 2015; Talhinhas et al., 2017). The pathogen prefers a temperature range of 20°C–28°C, requires a leaf wetness period only during spore germination, and penetrates the stomata of the host with germination hyphae (Hindorf and Omondi, 2011). Initially, the disease was less severe at elevations above 1,200 m above sea level (masl), where the environment was less conducive to rust attack (Zambolim, 2016). However, it has been reported to have expanded its distribution to higher altitudes, above 1,600 masl (Avelino et al., 2015). The fungus tolerates longer seasons without rainfall, only attacking leaves and not requiring another host to complete its life cycle (Hindorf and Omondi, 2011). The symptoms of CLR appear on the lower face of the leaf as large orange spore masses, leading to premature leaf defoliation and even plant death (Sousa et al., 2017; Koutouleas, 2023). This can reduce crop yield by 35% to 50% (Talhinhas et al., 2017; Zambolim, 2016), and in severe cases, yield losses can exceed 75% (Bebber et al., 2016; Koutouleas, 2023).

Effective management of CLR is of utmost importance for sustained production and productivity of coffee. The inclusion of resistant varieties is a cost-effective, ecosystem-friendly, and sustainable strategy for coffee leaf rust management (Talhinhas et al., 2017; Várzea et al., 2023). The resistance of the varieties depends on the genetic composition of both the pathogen and the variety itself. Currently, over 55 races of coffee leaf rust have been identified from different regions of the world, each affecting various coffee genotypes differently (M. do C. Silva et al., 2022). Resistant genes in Arabica coffee are designated as “S_H_ genes”. Among them, genes S_H_1, S_H_2, S_H_4, and S_H_5 have been identified within *C. arabica*, while the gene S_H_3 was introgressed from *C. liberica*, and S_H_6, 7, S_H_8, and S_H_9 were from *C. canephora* through the “Timor hybrids”, naturally occurring interspecific populations (Silva et al., 2022). Current commercial Arabica coffee varieties contain different combinations of a few of these known genes, suggesting significant variation in resistance among commercial varieties. It is likely that a single variety is not resistant to all the pathogen races (Zambolim and Caixeta, 2019). Furthermore, the pathogen continuously evolves; thus, varieties that show high resistance may eventually lose their ability to withstand it, highlighting the need to continuously search for and integrate new sources of resistance (McCook and Vandermeer, 2015).

The development of Arabica coffee varieties is concentrated in a few established breeding programs, but these varieties have been dispersed across various coffee-growing regions. However, when these varieties are introduced to other countries or continents, they often face environmental conditions different from those under which they were originally developed, potentially affecting their performance. To address this problem, the solution was to evaluate multiple varieties from different global breeding programs across multiple international locations. Multilocation trials allow for the identification of varieties with both broad and site-specific resistance, as well as sites with similar performance (Barrera et al., 2024; Olivoto et al., 2019). In recognition of that, World Coffee Research (WCR) initiated a program known as the International Multilocational Varieties Trial (IMLVT) in 2015. The IMLVT program engaged multiple global breeding programs to pool resources and knowledge to address coffee rust on an international scale. For this project, a first-of-its-kind trial, 31 Arabica coffee varieties from 11 coffee breeding programs around the world—most of which had never been tested on a broad basis—were evaluated at 29 sites in 18 coffee-growing countries. The objectives of this study were (1) to assess the global variety performance and stability of resistance to coffee leaf rust; (2) to assess the magnitude of the genotype-by-environment interaction; (3) to identify macroenvironments among the sites; and (4) to identify sites with high discriminant capacity.

## 2 Materials and methods

### 2.1 Sites

**Figure 1:**
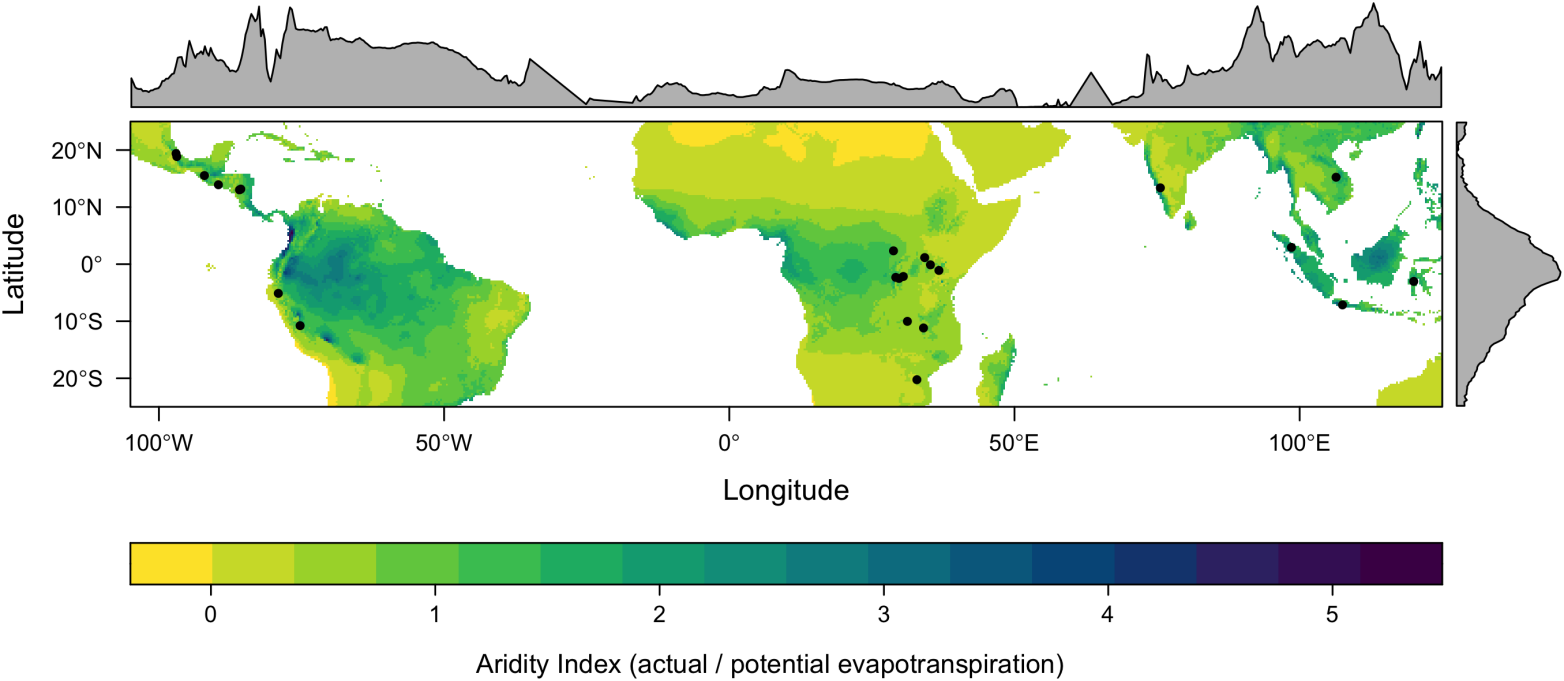
Location of the 23 study sites. The map shows the Priestley–Taylor α coefficient Aridity Index, while the marginal plots, representing the longitudinal and latitudinal averages, were generated using the rasterVis package (Perpinán and Hijmans, 2023).

From the sites established in the IMLVT located in Central and South America, Africa, and Asia, various sites were discarded due to a low number of varieties grown or less than 2 years of data, resulting in a total of 23 sites (**Figure 1**). These sites cover a wide range of climatic conditions, with minimal, maximal, and mean annual rainfall of 858, 3,795, and 1,792 mm, respectively; minimal, maximal, and mean temperatures of 17.1°C, 25.9°C, and 20.3°C, respectively; and minimal, maximal, and mean altitudes of 398, 1,931, and 1,297 masl, respectively (**Supplementary Table S1A**). Climatic data were taken from the WorldClim database (Fick and Hijmans, 2017) and the Global Aridity Index and Potential Evapotranspiration (Trabucco and Zomer, 2019).

### 2.2 Varieties

This study involved 29 varieties collected from 11 breeding programs in nine countries (**Supplementary Table S1B**). Two varieties were discarded from the original 31 IMLVT varieties due to low representativity across sites. The varieties exhibited two different growth habits—tall and dwarf—and came from different genetic groups: pure Arabicas (domesticated, core Ethiopia, Ethiopian legacy), Timor Hybrid-derived (catimor, sarchimor, cavimor, catucaí), and those with Liberica and Arabusta introgressions (Montagnon et al., 2022), as well as *F*_1_ hybrid varieties derived from Timor Hybrid lines and core Ethiopians. Agronomic practices followed the local protocols, which in most cases included some fungicide applications. The experimental design was a randomized complete block design (RCBD), with three blocks and 10 plants per block as the experimental unit.

### 2.3 Phenotyping

Plants were visually scored on a 1 to 5 scale (CLRs) based on Sera et al. (2010) to assess the plot-level resistance, ranging from no symptoms to full defoliation. A score of 1 indicates the absence of rust spots; 2, some rust spots without sporulation; 3, some rust spots with sporulation on some leaves; 4, the majority of leaves with rust spots with sporulation and some defoliation; and 5, the majority of leaves with rust spots with sporulation and heavy defoliation. Measurements were taken once or twice per year at peak rust pressure, from the vegetative stage through production. Scores were taken either as a representative value for the block or individually for each tree within the block. In the latter case, the average of the trees per block was computed. A total of 1,581 block–variety–site combination data points were included in the analyses.

### 2.4 Statistical analyses

A mixed model approach was used with the package Metan (Olivoto and Lúcio, 2020; Olivoto et al., 2019). In the model, the site and block within the site were treated as fixed effects, while genotype and the interaction of genotype-by-site were treated as random effects. Significance of effects, best linear unbiased predictor (BLUP), and genetic parameters were estimated. Broad-sense heritability was calculated as the proportion of genotypic variance to the sum of the genotypic variance, interaction variance, and residual variance. The coefficient of determination of the interaction effect was calculated as the proportion of the interaction variance to the sum of the genotypic variance, interaction variance, and residual variance. Heritability on the mean basis is calculated similarly to broad-sense heritability, except that the interaction variance is divided by the number of sites and the error variance is divided by the product of the number of sites and the number of blocks. The genotypic coefficient of variation is the square root of the genotypic variance divided by the mean. A genotype plus genotype-by-environment interaction (GGE) “Which-won-where” model biplot was used to evaluate the variety and identify mega-environments. The model is derived from nonscaled, environment-centered, symmetrical singular value decomposition (Yan et al., 2000). For GGE analyses, the data were imputed to fill the genotype-by-environment (GxE) matrix and inverted so that the winners are the genotypes with low scores. A weighted average of the absolute scores (WAASB) model, using the same mixed model formula, was deployed to assess the stability of varieties and discrimination capacity across sites. It uses singular value decomposition of the BLUPs for genotype-by-environment interaction effects (Olivoto et al., 2019).

## 3 Results

### 3.1 Sources of variation

The trial represents an unprecedented, global-scale effort to evaluate Arabica coffee varieties against natural infections of coffee leaf rust under diverse field conditions, testing 29 varieties in 23 sites in Central and South America, Africa, and Asia. By assessing the effects of variety, site, and their interaction, we found that all three factors, as well as the block within the site, were highly significant (**Table 1**). Broad-sense heritability, representing the proportion of variation accounted for by the genotype effect, was 0.31, while heritability on a mean basis was 0.94. The coefficient of determination for the interaction effects, indicating the proportion of variation explained by the genotype-by-site interaction, was 0.46. The genotypic coefficient of variation was 19.2%, and the overall mean rust score was 1.61 (**Table 1**).

**Table 1:** Likelihood ratio test, *F*-values, and genetic parameters of the model for coffee leaf rust scores of 29 varieties grown across 23 sites.

Parameter	Description	Value
LRTg	Likelihood ratio test—genotype	172.16^***^
LRTge	Likelihood ratio test—genotype × site	644.86^***^
Fe	*F*-value—site	21.49^***^
Fe:b	*F*-value—block within the site	2.749^***^
H2	Broad sense heritability	0.31
H2mg	Heritability on the mean basis	0.94
GElr2	Coefficient of determination of the interaction effects	0.46
CVg	Genotypic coefficient of variation	19.22
Mean	The overall mean of the CLR score	1.61

*** Indicates a p-value that is less than or equal to 0.001 (p ≤ 0.001).

### 3.2 Variety performance

**Figure 2:**
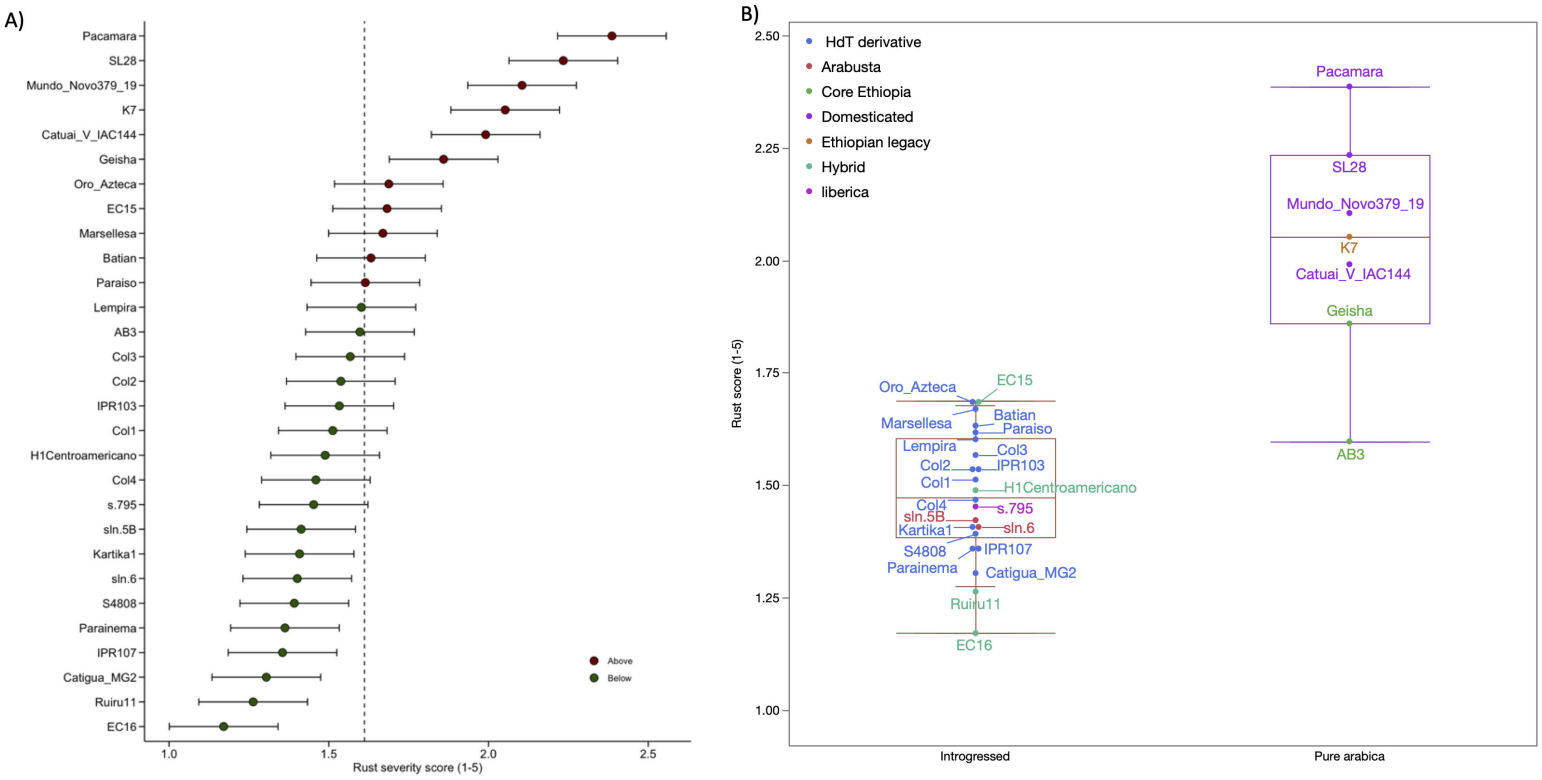
Performance of the varieties across sites. **(A)** Best linear unbiased prediction (BLUP) and 95% confidence interval for rust scores of the varieties in the IMLVT. Red and green circles represent the genotypes with BLUP values above and below the overall mean, respectively. Lower rust scores indicate higher resistance to rust. **(B)** Boxplot of rust score BLUPS by genetic group (introgressed, pure arabica), colored by subgroup.

**Figure 3:**
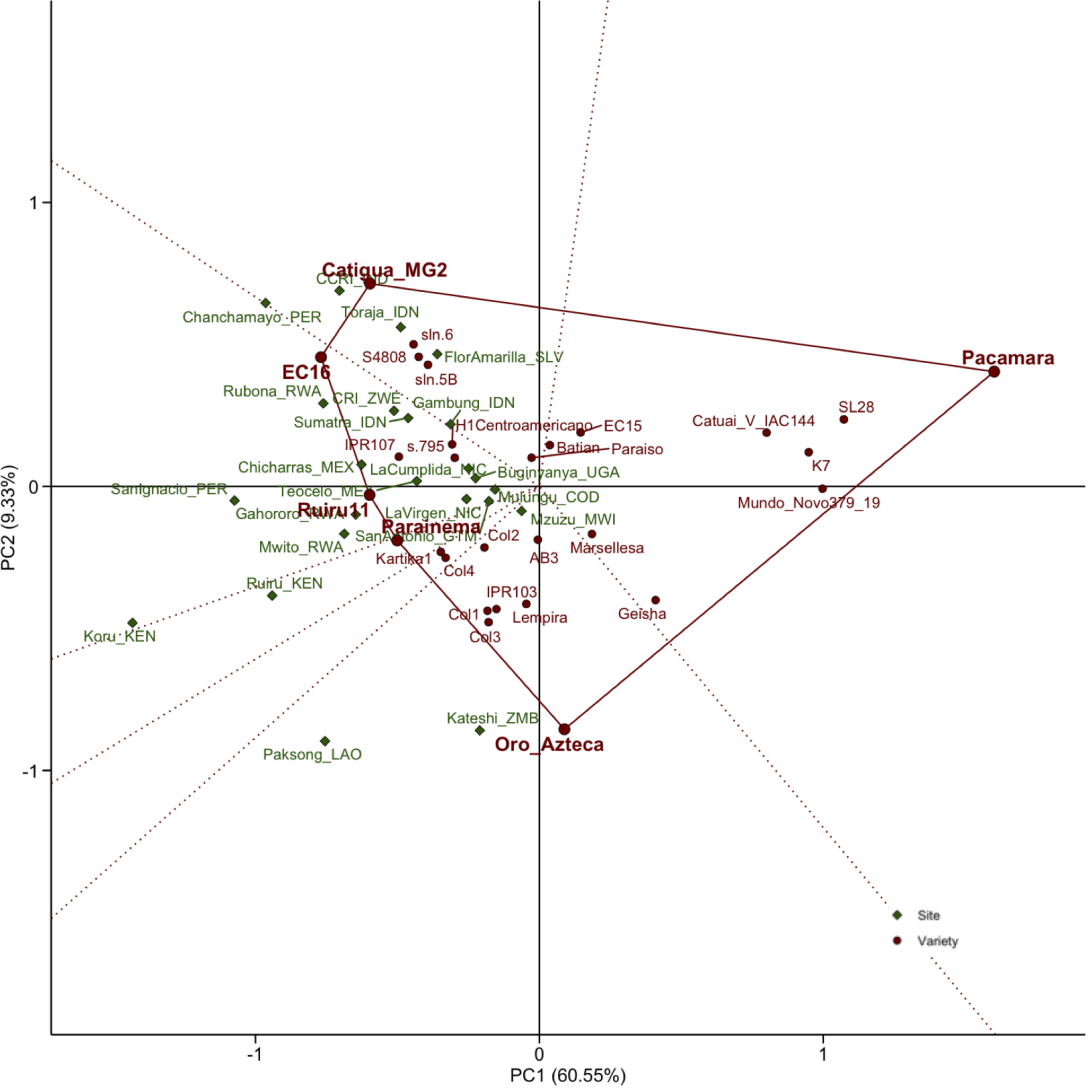
Genotype plus genotype-by-environment interaction (GGE) biplot (“Which-won-where”). The polygon includes the vertex genotypes (in bold), which represent the most responsive (resistant) varieties (in red) within site sectors (in green) divided by dotted lines.

While some varieties exhibited low scores (high resistance), no variety was immune, and all showed some rust symptoms at certain sites (**Figure 2A**). The variety with the lowest CLR score across sites (i.e., the most resistant to rust) was EC16, a hybrid commercially known as Mundo Maya, with a score of 1.12, while the variety with the highest score (more susceptible) was Pacamara, with a score of 2.43. Following EC16, the next most resistant varieties across sites were Ruiru11, Catigua MG2, IPR107, Parainema, S4808, and sln.6. The IMLVT varieties are derived from two main genetic backgrounds: pure Arabica and varieties with interspecific genetic introgressions. Although the IMLVT evaluation was not designed to test differences between and within genetic backgrounds, a comparison between the two sources, using a simple linear model, showed significant differences (*F* ratio = 54.4, *p* < 0.0001; **Figure 3**). Introgressed varieties had a mean CLR score of 1.47 (95% CI = 1.40–1.55), while the pure Arabicas had a mean CLR score of 2.03 (95% CI = 1.9–2.17). Interestingly, one pure Arabica from the core Ethiopia subgroup, AB3, had as low a score as some of the introgressed varieties (**Figure 2B**). The four F_1_ hybrid varieties were dispersed across the introgressed group, ranging from the most (EC16) to the least (EC15) resistant, with H1 and Ruiru 11 falling in between. The Arabustas and Liberica introgressed were not particularly resistant compared to the other Timor hybrid derivatives (**Figure 2B**).

### 3.3 Genotype plus genotype-by-environment interaction

The genotype plus GGE model was used r to understand GxE interactions and identify site groupings as well as the performance and responsiveness of specific varieties. The first two principal components associated with the CLR score accounted for 60.5% and 9.3% of the variation for principal component (PC)1 and PC2, respectively (**Figure 3**). **Figure 3** presents the “Which-won-where” biplot analysis, with vertex genotypes indicated in the polygon (Catigua MG2, EC16/Ruiru11, Parainema, Oro Azteca, and Pacamara). These vertex varieties represent the most responsive (resistant) varieties relative to the sites contained within the sectors, which are separated by the dotted red lines. Genotypes located near the origin would have similar rankings across environments and, thus, would not be particularly responsive. For example, the Catigua MG2 sector includes sites in India, El Salvador, Indonesia, and Peru. This sector also contains other varieties (S4808, sln.6, sln.5B) that performed similarly with higher resistance in these specific sites but were less responsive than Catigua MG2. The EC16/Ruiru11 sector includes the largest number of sites, located in Africa, Asia, and the Americas, while the Parainema sector includes only the Ruiru site in Kenya. The Oro Azteca sector includes three sites: the two southernmost sites in Africa and a site in Laos in India. Interestingly, this sector also includes other Catimor varieties (Lempira, Col1, Col2, and Col3). The Pacamara sector did not include any site, as the variety was consistently susceptible across all locations.

### 3.4 Stability analyses

**Figure 4:**
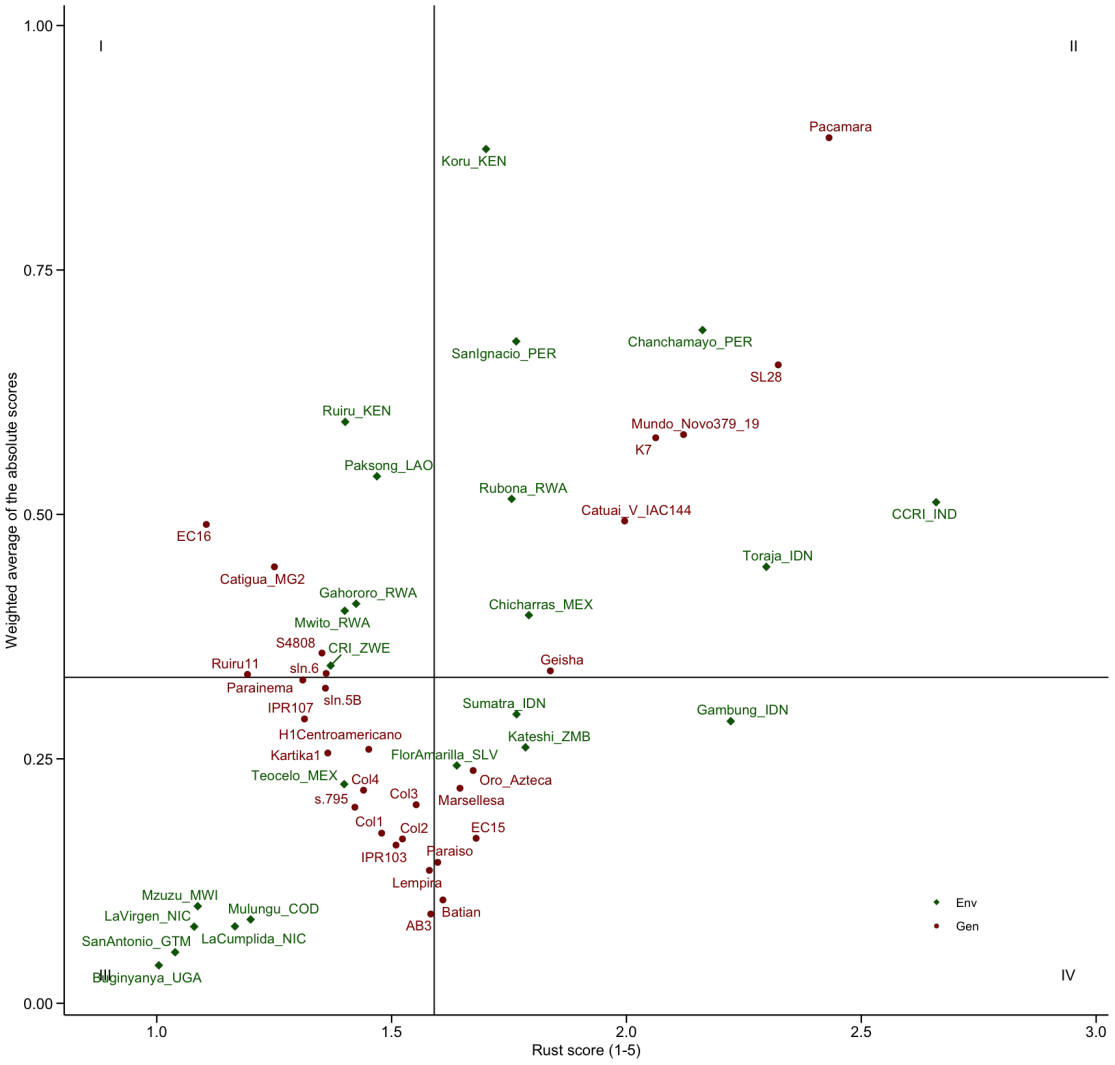
Biplot of the weighted average of the absolute scores (WAASB) estimating stability and overall rust score. Sites are color-coded in green, while varieties are color-coded in red.

The analysis using the WAASB model was employed to estimate stability in both genotype and site effects. Low WAASB scores indicate high stability for genotypes and low discriminatory ability for the sites. As shown in the biplot in **Figure 4**, which combines WAASB scores and overall performance, there is a positive association between stability scores and performance for both genotypes (*r* = 0.58) and sites (*r* = 0.57). For genotypes, this means that, in general, the more susceptible the genotype, the lower its stability. The results showed that it is possible to identify varieties with both high stability (low WAASB score) and high resistance (low rust score), such as Parainema, Kartika1, and IPR107, which were located in quadrant III. In contrast, varieties in quadrant I exhibited high resistance but low stability, including EC16, Catigua MG2, and Ruiru 11. For sites, the positive association between stability scores and performance means indicates that higher rust pressure enhances the ability to discriminate among varieties in terms of resistance. Nevertheless, a medium level of rust pressure appears adequate for effective field screening. The most discriminant sites—Koru (Kenya), Chanchamayo (Peru), San Ignacio (Peru), and Ruiru (Kenya)—had average levels of CLR scores, whereas the sites with the highest CLR scores—CCRI (India), Toraja (Indonesia), and Gambung (Indonesia)—showed less discriminant capacity.

## 4 Discussion

In this first-of-a-kind global cooperation of the multienvironmental evaluation of a diverse array of Arabica coffee varieties, resistance to coffee leaf rust was one of the key traits assessed due to its economic importance. The main objectives of this study were to assess the variation and performance of elite varieties for coffee leaf rust resistance across different environments and to evaluate the genotype-by-environment interaction. Both genotype and genotype-by-site interaction were highly significant, but the interaction explained a significantly higher proportion of the variation, suggesting that the expression of resistance (or susceptibility) levels can change according to the specific conditions of a site. The significant role of GxE was evident in the relatively medium level of broad-sense heritability; nevertheless, the high heritability on a mean basis suggests that the evaluation of CLR resistance in the field is reliable (Holland et al., 2003).

### 4.1 Variety performance

The results of the variety evaluation reveal a spectrum of rust resistance among the evaluated lines. None of the varieties exhibited complete immunity, underscoring the ongoing challenge of achieving high and durable rust resistance. Nevertheless, varieties with relatively high levels and stable resistance were identified, suggesting their potential for deployment in rust-prone regions. As expected, the varieties with a background of pure Arabica showed higher rust scores (more susceptibility) than the interspecific introgressed varieties, consistent with prior findings (Silva et al., 2006; Castro Caicedo et al., 2013; Eskes, 1983; Gichuru et al., 2021). However, there was variation within groups, with the noteworthy relatively high resistance performance of AB3, a line that belongs to the core Ethiopia group. Variations within wild and traditional varieties of indigenous trees in Ethiopia have shown differences in resistance to CLR (Chala et al., 2011), and it is known that the level of susceptibility of pure arabicas is dependent on the specific rust races (Rodrígues et al., 1976). It is possible that the AB3 could have a combination of the known genes from Arabica (S_H_1, S_H_2, S_H_4, S_H_5), or novel genes that have been selected in the rust-prone environment of Indonesia. Furthermore, AB3 has been identified as a parent of high-yielding populations (Akbar et al., 2022).

### 4.2 Identification of macroenvironments, specific variety performance, and stability

The “Which-won-where” genotype plus genotype-by-environment interaction biplot analysis identified four site sector clusters (macroenvironments). Different interspecific introgressed varieties were the most resistant and responsive in each sector, further suggesting that no single variety is optimal across all environments, and that selection should be conducted locally. Nevertheless, varieties such as Parainema, Kartika1, and IPR107 exhibited relatively high resistance and stability, meaning that while they may not be the most suitable for a specific site, they represent safer options for broader deployment. For specific performance, the sector with the highest number of sites identified EC16 and Ruiru11 as the most resistant varieties, which were also the two most resistant across all sites. The other three sectors, with fewer sites, identified Catigua MG2, Parainema, and Oro Azteca as their most responsive varieties. Interestingly, the sector where Oro Azteca was the vertex variety also included other Catimor cultivars, which, in general, exhibited the lowest resistance among the introgressed varieties. Previous research has indicated that in Central America and Brazil, the resistance of Catimors has been overcome (Brenes et al., 2025; Capucho et al., 2012). Therefore, it is possible that the three sites clustered in this sector, located in Africa and Asia, may have rust races that do not infect Catimors. The Catigua MG2 sector, which contains the site in India, also included three of the four varieties developed in India (S4808, sln.6, sln.5B). This suggests a degree of local adaptation, where these varieties were selected under the pressure of the local environment, agronomic practices, and pathogen diversity. The stability analysis showed variation in discriminant capacity across sites, indicating that some sites were better able to differentiate which varieties were more resistant to CLR than others. Taken together with the GGE results, within each macroenvironment, the most discriminant site could be deployed for the selection of rust resistance to optimize resources. For example, the CCRI site in India for the Catigua MG2 site sector, Koru in Kenya or the Peruvian sites for the broad EC16/Ruiru11 sector, and the Kateshi site in Zambia for the Oro Azteca sector. However, in general, the results showed that a moderate to high level of rust pressure is needed to better evaluate varieties.

### 4.3 Factors affecting GxE

The identification of mega-environments in the GGE and significant GxE effects in the mixed models suggest that, although much of the GxE variation could be statistically driven by highly susceptible varieties grown in low rust pressure sites, both environmental groups include sites with both high and low overall rust scores. Other factors should be considered and further studied, such as differences in the pathogen across the different sites. Previous studies have found differences in rust lineages between diploid and tetraploid species hosts (Silva et al., 2018; Rodrigues et al., 2022), as well as between and within geographic regions of *C. arabica* production (Ramírez-Camejo et al., 2022; Le et al., 2022; Rodrigues et al., 2022). Differences in agronomic practices, such as fertilization (Pérez et al., 2019; Merle et al., 2020), can result in more vigorous and healthy plants that show lower rust infection (Toniutti et al., 2017), but they can also lead to higher yield productivity and bienniality. This is because there is a positive correlation between rust damage and yield productivity (Eskes and Carvalho, 1983), with lower disease symptoms in low-yield years (Zambolim, 2016). Furthermore, the agricultural landscape (canopy arrangement, composition, and management) has also been shown to affect rust pressure (Avelino et al., 2023; Merle et al., 2020). In addition, geographic differences in the presence and diversity of beneficial microorganisms (Zewdie et al., 2021), as well as interactions at different trophic levels (e.g., hyperparasites–arthropod interactions) (Perfecto et al., 2014) should be considered. Finally, the effect of climate on the pathogen itself and its ability to infect the coffee host should be examined. The climate is changing at a rapid pace, particularly with increasing temperatures and humidity (Ayalew et al., 2024), which can lead to faster CLR incubation periods (Alfonsi et al., 2019; Ghini et al., 2011). Global and regional models predict an overall decrease in suitability for coffee production, with CLR being one of the negative factors (Torres Castillo et al., 2020; de Carvalho Alves and Sanches, 2022; Bilen et al., 2022). Breeding and the deployment of highly resistant varieties will continue to be crucial tools for ensuring coffee production and sustainability.

### 4.4 Conclusions

The importance of cooperation in coffee research and early breeding has enabled the evaluation of a diverse set of varieties across sites worldwide. The IMLVTs facilitated the identification of varieties with overall high rust resistance, as well as those with high stability or, alternatively, lower stability but strong performance under specific conditions. The latter, along with the identification of four main mega-environments, suggests the need for local testing prior to release and targeted breeding efforts tailored to specific conditions. Interspecific introgressed varieties and a pure Arabica genotype without introgression demonstrated high CLR resistance. Further studies are needed to identify the climatic and agronomic variables and conditions involved in genotype-by-environment interactions and to evaluate potential resistant coffee varieties in hotspot areas for CLR infection. Finally, continuous surveillance is essential to monitor the pathogen’s spread, identify new mutations, assess resistance breakdown in commercial varieties, discover new sources of resistance, and rapidly incorporate both known and novel resistances into breeding programs. Combining multiple resistance genes could provide “an efficient barrier against new race formation of the pathogen”, as proposed by Eskes et al. (1990).

## Data Availability

The original contributions presented in the study are included in the article/**Supplementary Material**. Further inquiries can be directed to the corresponding author.
